# Long‐term risk of cardiovascular mortality in lymphoma survivors: A systematic review and meta‐analysis

**DOI:** 10.1002/cam4.1572

**Published:** 2018-08-15

**Authors:** Devon J. Boyne, Alexis T. Mickle, Darren R. Brenner, Christine M. Friedenreich, Winson Y. Cheung, Karen L. Tang, Todd A. Wilson, Diane L. Lorenzetti, Matthew T. James, Paul E. Ronksley, Doreen M. Rabi

**Affiliations:** ^1^ Department of Cancer Epidemiology and Prevention Research Cancer Control Alberta Alberta Health Services Calgary AB Canada; ^2^ Department of Community Health Sciences Cumming School of Medicine University of Calgary Calgary AB Canada; ^3^ Department of Oncology Cumming School of Medicine University of Calgary Calgary AB Canada; ^4^ Department of Medicine Cumming School of Medicine University of Calgary Calgary AB Canada; ^5^ Department of Cardiac Sciences Cumming School of Medicine University of Calgary Calgary AB Canada

**Keywords:** cardiovascular disease, Hodgkin, lymphoma, meta‐analysis, mortality, standardized mortality ratio, survivors, systematic review

## Abstract

Cardiovascular disease has been identified as one of the late complications of cancer therapy. The purpose of this study was to quantify the long‐term risk of cardiovascular mortality among lymphoma survivors relative to that of the general population. A systematic review and meta‐analysis were conducted. Articles were identified in November 2016 by searching EMBASE, MEDLINE, and CINAHL databases. Observational studies were included if they assessed cardiovascular mortality in patients with lymphoma who survived for at least 5 years from time of diagnosis or if they had a median follow‐up of 10 years. A pooled standardized mortality ratio (SMR) was estimated using a DerSimonian and Laird random‐effects model. The *Q* and *I* ^2^ statistics were used to assess heterogeneity. Funnel plots and Begg's and Egger's tests were used to evaluate publication bias. Of the 7450 articles screened, 27 studies were included in the systematic review representing 46 829 Hodgkin and 14 764 non‐Hodgkin lymphoma survivors. The pooled number of deaths attributable to cardiovascular disease among Hodgkin and non‐Hodgkin disease was estimated to be 7.31 (95% CI: 5.29‐10.10; *I* ^2^ = 95.4%) and 5.35 (95% CI: 2.55‐11.24; *I* ^2^ = 94.0%) times that of the general population, respectively. This association was greater among Hodgkin lymphoma survivors treated before the age of 21 (pooled SMR = 13.43; 95% CI: 9.22‐19.57; *I* ^2^ = 78.9%). There was a high degree of heterogeneity and a high risk of bias due to confounding in this body of literature. Lymphoma survivors have an increased risk of fatal cardiovascular events compared to the general population and should be targeted for cardiovascular screening and prevention campaigns.

## 1 BACKGROUND

The success and continued progress of cancer control strategies have resulted in a rise in the number of long‐term cancer survivors.^1^, ^2^ This growing patient population brings with it a set of unique healthcare needs that are becoming increasingly apparent. Mounting epidemiologic evidence suggests that individuals previously treated for cancer have an increased risk of several adverse health outcomes later in life which include secondary cancers, cardiovascular disease, fertility issues, sexual dysfunction, endocrine disorders, neurocognitive impairment, chronic fatigue, and various psychosocial problems.^3^, ^4^, ^5^, ^6^, ^7^, ^8^, ^9^, ^10^, ^11^, ^12^, ^13^, ^14^, ^15^, ^16^ Thoroughly understanding these late effects can help to inform ongoing efforts to develop and implement prevention and screening programs aimed at managing and mitigating the burden of chronic disease associated with cancer and its treatment.^17^, ^18^, ^19^, ^20^, ^21^, ^22^, ^23^, ^24^, ^25^

Hodgkin and non‐Hodgkin lymphoma are hematological malignancies common in both children and adults.^1^, ^2^ Advances in cancer treatment have led to improved survival rates for both Hodgkin and non‐Hodgkin lymphoma patients with current 5‐year survival estimates at approximately 85% and 65%, respectively.^1^, ^2^, ^26^, ^27^, ^28^, ^29^ Treatment for lymphoma typically involves chemotherapy alone or in combination with radiation, stem cell transplantation, or biologic therapies.^1^ Although efficacious in the control of lymphoma, some of these therapeutic options are now recognized as causative agents in cardiovascular disease.^30^, ^31^, ^32^ Given the increasing rates of survival, the younger age of onset, and the potential for cardiotoxic treatment effects, quantifying the long‐term risk of cardiovascular mortality among patients with lymphoma is a timely and important task.

Although there have been systematic reviews and meta‐analyses investigating the risk of metabolic syndrome^33^ and the prevalence of cardiovascular disease^34^ in lymphoma survivors, the long‐term risk of fatal cardiovascular outcomes remains unknown.^35^, ^36^, ^37^, ^38^, ^39^, ^40^ We conducted a systematic review and meta‐analysis of the observational epidemiologic literature to quantify the risk of cardiovascular mortality among lymphoma survivors relative to the general population. We hypothesized that lymphoma survivors would have an increased risk of dying from cardiovascular disease compared to the general population.

## 2 METHODS

A protocol for this review was published in the International Prospective Register of Systematic Reviews (PROSPERO) database (registration number: CRD42016052342). This systematic review follows the reporting guidelines proposed by the Meta‐analysis of Observational Studies in Epidemiology (MOOSE) group.^41^

### 2.1 Search strategy and information sources

EMBASE, MEDLINE, and CINAHL were searched from inception to 22 November 2016. Our search strategy combined terms from 4 themes related to our research question: (1) lymphoma, (2) long‐term survivor; (3) cardiovascular disease; and (4) observational study. Terms were searched as both keywords (title/abstract words) and subject headings as appropriate. The observational study designs filter included in this search was adapted from 2 previously published filters.^42^, ^43^ No restrictions were placed on language or year of publication. A detailed description of this systematic search strategy can be found in Table S1. Additional articles were identified by screening the references of the eligible studies identified from the database search and by manually searching top‐tiered journals that publish epidemiologic studies on cancer or heart disease. This search strategy was re‐run on 7 November 2017 to ensure that it was up‐to‐date at the time of manuscript submission.

### 2.2 Eligibility criteria

An assessment of study eligibility was independently undertaken by 2 reviewers in duplicate (DJB and AM). Eligibility was assessed in a two‐stage process. In the first stage, the title and abstracts of each study were screened. Studies were considered for full‐text review if they met all of the following criteria: (1) the study was published in a peer‐reviewed journal; (2) original data were presented; (3) human participants were under investigation; (4) the article was relevant to the objectives of this review. In the second stage, the remaining studies were assessed in their entirety. To be included in this review, an investigation had to satisfy all of the following criteria: (1) the population studied were patients with a diagnosis of and prior treatment for lymphoma; (2) the patients survived for a minimum of 5 years after diagnosis, the study had a median follow‐up of at least 10 years from the time of diagnosis, or the study presented risk estimates specific to individuals who survived for 5 years or more after their diagnosis; (3) there was a comparator group that was representative of the general population; (4) the outcomes reported included standardized mortality, risk, hazards, or odds ratios, or sufficient data were provided for their calculation; (5) the study was of a cohort, case‐control, nested case‐control, case‐cohort, or cross‐sectional design.

Agreement between the 2 reviewers was quantified using percent agreement and kappa statistics. Disagreements were resolved by consensus. In situations where 2 or more eligible studies were conducted on the same study population, the study with the largest sample size was retained in the review and those with smaller sample sizes were excluded.

### 2.3 Data extraction and study quality assessment

Data from eligible studies were extracted using a predefined data template. For each study, data regarding the study population (sex, median age at diagnosis, and lymphoma staging), study characteristics (country, median duration of follow‐up, number of survivors, and number of events), and treatment regimen (proportion receiving anthracycline chemotherapy, proportion receiving mantle field radiation, and treatment era) were extracted. When median values were not reported for relevant variables (age at diagnosis and duration of follow‐up), mean values were used. Study quality was assessed by a single reviewer using the Newcastle Ottawa Scale which ranges from 0 (low quality) to 9 (high quality) and appraises studies across 3 domains: (1) the selection of participants; (2) the control of confounding; (3) and the assessment of outcomes.^44^

### 2.4 Statistical analysis

The primary outcome of interest was the pooled standardized mortality ratio (SMR) describing the observed number of deaths due to cardiovascular disease among lymphoma survivors relative to the expected number of deaths due to cardiovascular disease in the general population. Given the inherent heterogeneity in the population of patients represented by this body of literature, all meta‐analyses were conducted using a DerSimonian and Laird random‐effects model. In situations where a study reported stratified estimates, a Mantel‐Haenszel fixed effects model was used to estimate a single overall effect estimate for that study. A cumulative meta‐analysis was conducted to understand how emerging studies on the association between lymphoma and treatment exposure and cardiovascular mortality changed the pooled estimate over time. Stratified meta‐analyses and meta‐regression were also conducted across strata defined by age at diagnosis, sex, treatment era, duration of follow‐up, and treatment regimen. The standard error (SE) of the log‐transformed SMR or hazard ratio (HR) was estimated using the following formula: SE (log HR) or SE (log SMR) = (log upper confidence interval − log lower confidence interval)/3.92.^45^

The degree of heterogeneity in the literature was assessed using the *Q*‐ and *I* ^2^‐statistics in tandem with a visual examination of the forest plots. Sources of heterogeneity were identified using subgroup analyses and meta‐regression. Publication bias was assessed qualitatively through a visual inspection of a funnel plot and quantitatively using Begg's rank correlation test and Egger's regression test for funnel plot asymmetry. The trim‐and‐fill method was used to explore the robustness of our results to publication bias. All analyses were carried out using the *meta* and *metafor* packages in RStudio version 1.0.143 with the exception of the subgroup and meta‐regression analyses which were performed using the *metan* and *metareg* commands in Stata version 14.2.

## 3 RESULTS

### 3.1 Study characteristics

**Figure 1:**
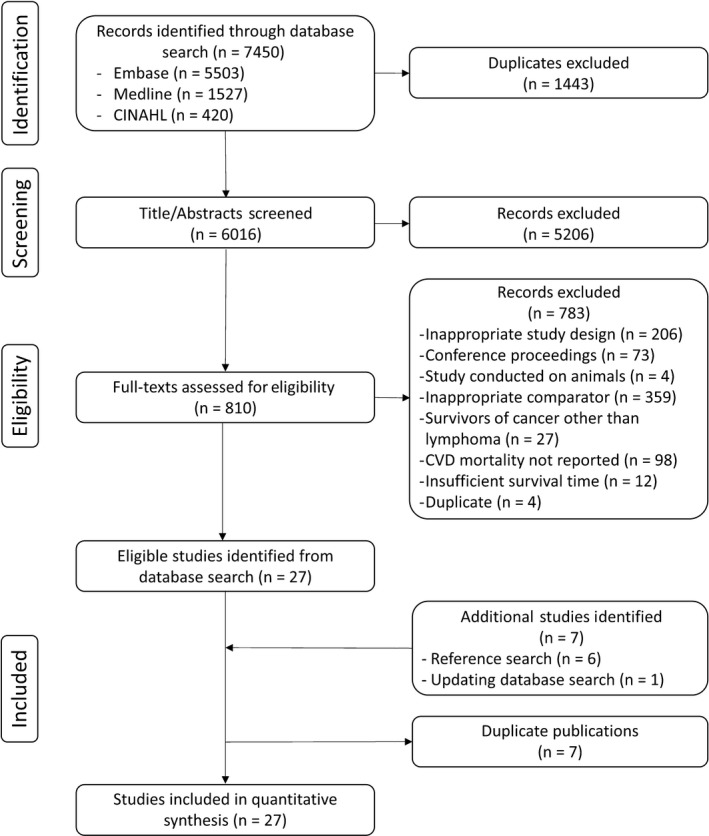
PRISMA flow diagram

The database search resulted in the identification of 7450 articles of which 27 were deemed to be eligible for inclusion (Figure 1).^46^, ^47^, ^48^, ^49^, ^50^, ^51^, ^52^, ^53^, ^54^, ^55^, ^56^, ^57^, ^58^, ^59^, ^60^, ^61^, ^62^, ^63^, ^64^, ^65^, ^66^, ^67^, ^68^, ^69^, ^70^, ^71^, ^72^ There was strong agreement between the 2 reviewers at both stages of the eligibility assessment. The percent agreement and the kappa statistics respectively were 93.2% and 0.62 for the first stage and 97.0% and 0.73 for the second stage.

In total, the evidence base consisted of 46 829 Hodgkin and 14 764 non‐Hodgkin lymphoma survivors treated between 1940 and 2006 who experienced a total of 1236 and 208 fatal cardiovascular events, respectively (Table 1). All studies were of a cohort design, and all reported a standardized mortality ratio except for the study by Kiserud et al,^67^ which presented a hazard ratio. Of the 27 articles included in this review, 12 (44.4%) originated from North America. The median duration of follow‐up was 13.8 years (range: 9.5‐24.3; IQR: 11.2‐18.2), median age at treatment was 25.8 years (range: 6.6‐56.8; IQR: 19.2‐31.1), and the percent of females in the study population was 45.1% (range: 0.0‐61.8; IQR: 42.6‐50.6).

**Table 1:** Study characteristics of articles included in systematic review (by year of publication, n = 27)

First author (year)	Cohort Designation	Country	Hodgkin		Non‐Hodgkin		Female (%)	Treatment Era (y)	Median age at diagnosis (y)	Median follow‐up (y)	Anthracycline exposure^a^ (%)	Mantle field radiation^b^ (%)
			Survivors (n)	Deaths (n)	Survivors (n)	Deaths (n)						
Henry‐Amar (1990)^46^	EORTC Lymphoma Cooperative Group^c^	Various	1449	17	—	—	43.0	1963‐1986	31.2	N/A	8.0	100.0
Hancock (1993)^47^	Stanford University Medical Center	USA	635	12	—	—	44.7	1961‐1991	15.4	10.3	12.9	83.8
Hancock (1993)^48^	Stanford University Medical Center	USA	2232	88	—	—	41.0	1960‐1995	29.0	9.5^d^	9.0	72.1
Robertson (1994)^49^	British National Register of Childhood Tumours	UK	726	1	450	0	N/A	1971‐1985	<15^e^	N/A	N/A	N/A
Mauch (1995)^50^	Harvard‐affiliated hospitals	USA	794	15	—	—	44.0	1969‐1988	24.0	11.0	5.4	99.6
King (1996)^51^	University of Rochester Hospital	USA	326	7	—	—	50.6	1954‐1989	25.6	13.3	N/A	100.0
Glanzmann (1998)^53^	University Hospital of Zurich	Switzerland	352	13	—	—	N/A	1964‐1992	33.8	11.2	26.7	100.0
Brierley (1998)^52^	Princess Margaret Hospital	Canada	611	14	—	—	45.7	1973‐1984	31.0	11.0	2.3	80.4
Hudson (1998)^54^	St. Jude Children's Research Hospital	USA	387	6	—	—	42.6	1968‐1990	14.4	15.1	28.2	68.0
Reinders (1999)^56^	Daniel den Hoed Cancer Center	Netherlands	258	12	—	—	47.7	1965‐1980	28.0	14.2	0.0	100.0
Shah (1999)^57^	St. Jude Children's Research Hospital	USA	106	3	—	—	41.5	1970‐1995	14.7	13.3	39.6	20.8
Green (1999)^55^	Roswell Park Cancer Institute	USA	58	2	30	1	0.0	1960‐1989	10.9	24.1	N/A	N/A
Eriksson (2000)^59^	Radiumhemmet Karolinska Hospital	Sweden	157	13	—	—	38.2	1972‐1985	33.0	16.0	45.2	N/A
Avilés (2000)^58^	National Medical Center Oncology Hospital	Mexico	2980	39	—	—	54.5	1970‐1995	14.6^f^	14.6	N/A	54.8
Lee (2000)^60^	University of Minnesota Hospital	USA	210	16	—	—	46.7	1970‐1986	25.8^f^	15.6	N/A	100.0
Avilés (2001)^61^	National Medical Center Oncology Hospital	Mexico	—	—	714	7	57.4	1975‐1995	56.8	N/A	91.6	19.3
Ng (2002)^62^	Harvard‐affiliated hospitals	USA	1080	17	—	—	45.7	1969‐1997	25.0	12.0	16.3	61.9
Aleman (2003)^63^	Netherland Hospitals	Netherlands	1261	45	—	—	42.7	1965‐1987	26.0	17.8	N/A	N/A
Avilés (2005)^64^	National Medical Center Oncology Hospital	Mexico	476	20	—	—	54.0	1988‐1996	39.6	11.5	100.0	0.0
Swerdlow (2007)^65^	Collaborative British Cohort Study	UK	7033	166	—	—	38.1	1967‐2000	34.8^f^	9.9^c^	26.6	32.1
Mertens (2008)^66^	Childhood Cancer Survivor Study	USA	2717	62	1524	13	44.7	1970‐1986	7.8^f^	N/A	N/A	N/A
Kiserud (2010)^67^	Norwegian Radium Hospital	Norway	557	36	—	—	43.0	1971‐1991	30.0	13.0	26.6	78.0
Prasad (2012)^68^	Finnish Cancer Registry	Finland	1084	44	557	14	54.4	1966‐1999	19.2^f^	20.9	N/A	N/A
Kero (2015)^69^	Finish Cancer Registry	Finland	1693	75	923	27	43.7	1966‐2004	21.4^f^	N/A	N/A	N/A
Bhuller (2016)^70^	British Columbia Cancer Registry	Canada	442	8	—	—	50.0	1970‐1999	19.7^f^	19.6	N/A	N.A
Henson (2016)^71^	Teenage and Young Adult Cancer Survivor Cohort	UK	16 971	472	9467	129	61.8	1971‐2006	31.1^f^	19.3	N/A	N/A
Fidler (2017)^72^	British Childhood Cancer Survivor Study	UK	2234	33	1549	17	45.1	1940‐2006	6.6	23.0	N/A	N/A

a Primarily doxorubicin but also included epirubicin and mitoxantone.

b Including patients who received extended field or total nodal radiation.

c Study conducted on participants from 4 clinical trials conducted by the European Organization for Research and Treatment of Cancer (EORTC) Lymphoma Cooperative Group.

d Although the median follow‐up was less than 10 y, the study was included because it presented risk estimates stratified by follow‐up.

e Study did not report the median age at diagnosis, but all participants were diagnosed with lymphoma before the age of 15 y.

f Expected value calculated using available information.

### 3.2 Study quality assessment

The Newcastle Ottawa Scale score for each study is presented in Table S2. Most studies scored 6 points (n = 11; 40.7%) or 7 points (n = 11; 40.7%) of a possible 9 points on the Newcastle Ottawa Scale. No studies included in this review excluded individuals with a history of cardiovascular disease at baseline. Six studies did not have a representative cohort of lymphoma survivors as they were conducted on clinical trial participants. Every study controlled for age and sex, and 2 studies additionally controlled for ethnicity. Aside from age, sex, and ethnicity, no other potential confounders were adjusted for in any of the analyses. All studies had an adequate duration of follow‐up which we defined as 5 or more years since time of diagnosis. Five investigations did not report the way in which the outcome was captured; however, the outcome was objectively measured in the remaining studies. Fourteen studies did not describe the attrition of participants or had a loss‐to‐follow‐up greater than 5% with no description of the lost participants.

### 3.3 Meta‐analyses

**Figure 2:**
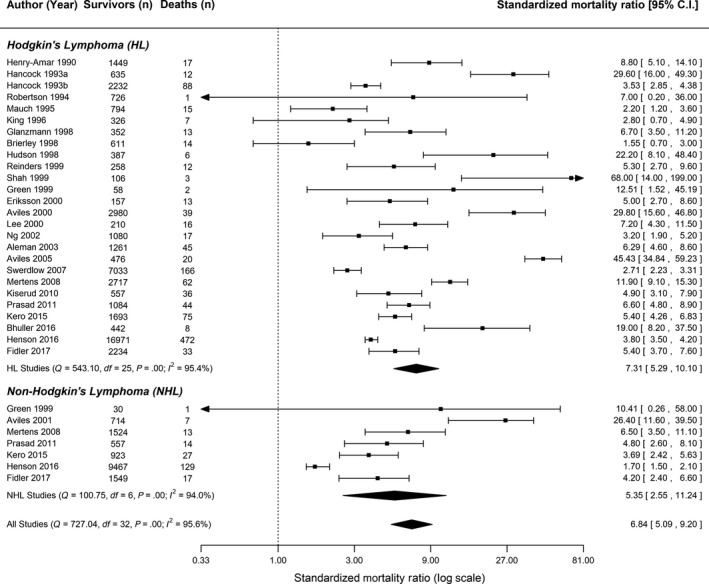
Forest plot of the long‐term risk of cardiovascular disease mortality among lymphoma survivors

The pooled number of deaths due to cardiovascular disease among Hodgkin and non‐Hodgkin lymphoma survivors was estimated to be 7.31 (95% CI: 5.29‐10.10) and 5.35 (95% CI: 2.55‐11.24) times greater than the expected number of deaths due to cardiovascular disease in the general population, respectively (Figure 2). The estimated pooled SMR for all lymphoma survivors was 6.84 (95% CI: 5.09‐9.20). There was no statistically significant difference in the pooled standardized mortality ratio for Hodgkin and non‐Hodgkin lymphoma survivors (meta‐regression *P*‐value = .43). As visualized in the forest plot of the cumulative meta‐analysis (Figure S1), studies have consistently reported an elevated risk of cardiovascular mortality among Hodgkin lymphoma survivors over time. Due to the limited number of investigations, a cumulative meta‐analysis of results from the non‐Hodgkin lymphoma studies was not conducted.

Meta‐analyses stratified by age at diagnosis (<21 years vs ≥21 years), sex (male vs female), duration of follow‐up (<10 years vs 10 to <15 years vs 15 to <20 years vs ≥20 years), treatment regimen (radiation only vs radiation and chemotherapy), and treatment era (<1980 vs ≥1980) were conducted (Table S3). Among Hodgkin lymphoma survivors who were diagnosed before the age of 21, the estimated SMR was 13.43 (95% CI: 9.22‐19.57) which was significantly different from the estimated SMR among Hodgkin lymphoma survivors who were 21 years of age or older at the time of diagnosis (pooled SMR = 3.33, 95% CI: 2.54‐4.35; meta‐regression *P*‐value = .001). This difference in the magnitude of effect by age at diagnosis was not observed in non‐Hodgkin lymphoma survivors (pooled SMR_age at diagnosis < 21 years_ = 6.23, 95% CI: 3.35‐11.59; pooled SMR_age at diagnosis ≥ 21 years_ = 6.61, 95% CI: 0.45‐96.59; meta‐regression *P*‐value = .97). Among Hodgkin lymphoma survivors, there was no statistically significant difference in the estimated SMRs by sex, duration of follow‐up, treatment regimen, or treatment era (meta‐regression *P*‐value > .35). These stratified meta‐analyses were not conducted among studies of non‐Hodgkin lymphoma survivors because of insufficient information.

### 3.4 Assessment of heterogeneity

The degree of heterogeneity in this evidence base was considerable (*Q* statistic < 0.01; *I* ^2^ statistic >94.0%). Potential sources of heterogeneity assessed through subgroup meta‐analyses and meta‐regression are presented in Table 2. Among studies of Hodgkin lymphoma, the median age at diagnosis (<21 years vs ≥21 years) and the percent of participants who had stage I or stage II lymphoma (<50% vs ≥50%) were statistically and clinically significant sources of heterogeneity (meta‐regression *P*‐value <.05). Among studies of non‐Hodgkin lymphoma, the maximum treatment era (<1997 vs ≥1997) and Newcastle Ottawa Scale selection score were identified as significant sources of heterogeneity (*P* < .05). Although not statistically significant, the magnitude of effect reported by studies with a total Newcastle Ottawa Scale of 7 (higher quality) tended to be smaller than studies with a total Newcastle Ottawa Scale of 6 or less (lower quality) for both Hodgkin and non‐Hodgkin lymphoma.

**Table 2:** Assessment of heterogeneity in investigations of the long‐term risk of cardiovascular mortality among lymphoma survivors

	Hodgkin (N = 26)			Non‐Hodgkin (N = 7)		
	N^b^	SMR (95% C.I.)^a^	*P*‐value^b^	N^b^	SMR (95% C.I.)^a^	*P*‐value^b^
Demographics						
Percent female						
<50%	18	6.17 (4.50‐8.48)	.99	3	5.15 (3.53‐7.50)	.58
≥50%	6	11.19 (3.83‐32.72)		4	5.17 (1.79‐14.99)	
Median age at diagnosis						
<21 years	8	14.10 (8.64‐23.01)	.006	4	5.04 (3.63‐6.90)	.96
≥21 years	16	4.96 (3.40‐7.24)		3	5.34 (1.36‐20.87)	
Percent stage I or II						
<50%	2	39.52 (26.79‐58.30)	.045	1	—	—
≥50%	9	5.91 (3.51‐9.96)		0	—	
Study characteristics						
Country						
North American	12	7.66 (4.42‐13.27)	.85	2	6.63 (3.77‐11.67)	.69
Other	14	7.12 (4.42‐13.27)		5	4.92 (2.09‐11.65)	
Median follow‐up						
<15 years	14	7.02 (3.61‐13.68)	.72	0	—	—
≥15 years	10	7.73 (5.29‐11.28)		5	—	
Number of survivors						
<1000 survivors	15	8.84 (4.58‐17.05)	.33	4	7.89 (2.75‐22.68)	.29
≥1000 survivors	11	5.92 (4.37‐8.02)		3	3.48 (1.44‐8.40)	
Number of deaths^b^						
<20 deaths	15	7.38 (4.56‐11.96)	.97	5	7.73 (3.66‐16.33)	.13
≥20 deaths	11	7.28 (4.56‐11.63)		2	2.44 (1.14‐5.21)	
Treatment Regimen						
Percent who received anthracyclines^b^						
<25%	7	7.60 (5.30‐10.92)	.19	0	—	—
≥25%	7	11.23 (3.64‐34.69)		1	—	
Percent who received mantle field radiation^b^						
<75%	7	12.29 (4.37‐34.58)	.17	1	—	—
≥75%	9	5.40 (3.13‐9.32)		0	—	
Maximum treatment era						
<1997	20	8.10 (5.08‐12.29)	.40	3	12.64 (3.89‐41.12)	.049
≥1997	6	5.13 (3.76‐6.99)		4	3.25 (1.80‐5.87)	
Newcastle Ottawa Scale						
Selection score						
3 points	22	6.82 (4.80‐9.71)	.40	6	3.86 (2.18‐6.84)	.03
2 points	4	10.90 (3.04‐39.04)		1	26.40 (14.31‐48.72)	
Comparability score						
2 points	2	3.49 (2.83‐4.31)	.20	0	—	—
1 point	24	7.85 (5.52‐11.16)		7	—	
Outcome score						
3 points	12	6.45 (4.53‐9.18)	.52	4	3.75 (1.80‐7.83)	.23
1 or 2 points	14	8.41 (4.55‐15.56)		3	9.86 (1.89‐51.46)	
Total score						
7 points	11	5.87 (4.17‐8.28)	.26	4	3.75 (1.80‐7.83)	.23
6, 5, or 4 points	15	9.05 (5.05‐16.21)		3	9.86 (1.89‐51.46)	

Number of studies in subgroup.

Pooled standardized mortality ratio estimate (95% confidence intervals) of study estimates specific to subgroup from random‐effects model.

*P*‐value corresponds to the significance of an indicator variable for subgroup in a meta‐regression model.

Deaths caused by cardiovascular disease.

a Primarily doxorubicin but also included epirubicin and mitoxantone.

b Including patients who received extended field or total nodal radiation.

### 3.5 Publication bias

There was no evidence of statistically significant publication bias according to Begg's test (*P*‐value = .30) or Egger's test (*P*‐value = .12). However, the presence of funnel plot asymmetry (Figure S2) suggested a potential lack of small studies reporting small effect sizes. Therefore, we carried out the trim‐and‐fill procedure. Using this method, the number of studies estimated to be missing was zero and the overall effect estimate was unchanged. When repeating these analyses for studies on Hodgkin and non‐Hodgkin lymphoma survivors separately, we similarly found a lack of evidence that would suggest publication bias (data not shown).

## 4 DISCUSSION

Our findings suggest that both Hodgkin and non‐Hodgkin lymphoma survivors have an elevated risk of experiencing a fatal cardiovascular event compared to the general population. This association is unlikely to be spurious according to Bradford Hill's criteria for causation.^73^ The magnitude of the estimated effect along with the consistency of the association and its established temporality argue strongly against a spurious association and suggest a possible causal relationship. Further, previous studies have found a dose‐response relation between the amount of chest radiation and the cumulative dose of anthracyclines with cardiovascular disease risk which provides support for a biologic gradient.^9^, ^74^, ^75^, ^76^ Lastly, there is a high degree of biological plausibility with respect to the relation of interest. Anthracyclines are established cardiotoxic agents which are thought to impact cardiovascular function through the production of reactive oxygen species and other biologic mechanisms.^32^ Radiation therapy is known to cause direct damage to the heart and surrounding vasculature.^30^ In addition, exposure to anthracyclines and chest radiation have been associated with intermediate endpoints on the causal pathway such as atherosclerosis and reduced left ventricle function.^77^, ^78^

There is evidence that individuals treated for Hodgkin lymphoma before the age of 21 may be at a particularly high risk of cardiovascular mortality. Our stratified meta‐analyses showed that there were statistically and clinically significant differences in the estimated SMR by age at diagnosis. This age difference in the susceptibility to the cardiovascular effects of cancer treatment has been described in previous reviews, and there exist several plausible mechanisms.^79^, ^80^ Cancer treatment may impair normal cardiovascular development among children and young adults which could partially explain the observed age differences in the estimated effect. Alternatively, a developing cardiovascular system may be more susceptible to the cardiotoxic insults of cancer treatment. This disparity may also reflect differences in treatment regimens as participants treated during childhood would have received treatment in an earlier era when the dose of anthracyclines and the prevalence of mantle field radiation were higher compared to more recent eras.^81^ Cancer treatment could also indirectly affect the risk of cardiovascular disease mortality by impacting various behavioral and psychosocial determinants of health.^82^, ^83^ These indirect pathways may play a lesser role in mediating the relation between lymphoma treatment and cardiovascular disease risk among those treated later in life which could partially account for this heterogeneity. Regardless of the underlying mechanism, our results suggest that childhood Hodgkin lymphoma survivors are a population with a particularly high risk of experiencing a fatal cardiovascular event.

There exists considerable heterogeneity in this body of literature. Both age and lymphoma stage at the time of diagnosis were clinically and statistically significant sources of heterogeneity. There was also a suggestion that treatment characteristics and study quality were important sources of heterogeneity. As expected, larger effect estimates were reported in studies with a greater proportion of participants who received anthracyclines and in studies conducted before 1997 (see Table 2). The larger effect estimates among studies conducted prior to 1997 may be attributable to increased anthracycline and mantle field radiation exposure, as previously described, or may be due to the longer duration of follow‐up.^81^ Although not statistically significant, investigations where a greater proportion of patients received mantle field, extended field, or total nodal radiation tended to report smaller effect estimates. The unexpected direction of this trend may be artifactual or due to confounding at the individual level. Across all domains of the Newcastle Ottawa Scale, there was a consistent trend that larger effect estimates were reported in lower quality studies. The main reasons for the lower study quality were the predominant inclusion of patients from clinical trials, the failure to adjust for ethnicity, and the potential for bias due to loss‐to‐follow‐up.

Given the limitations in the literature, 3 important caveats should be considered when interpreting the results from this meta‐analysis. First, there is a risk of bias in our pooled effect estimates due to failure to adjust for potential confounders such as tobacco use, the presence of diabetes, and other risk factors associated with both lymphoma and cardiovascular disease.^84^, ^85^, ^86^ As the individual studies did not adjust for cardiovascular disease risk factors aside from age, sex, and ethnicity, the estimated pooled SMR may have overestimated the true SMR of cardiovascular mortality in this study population. However, it is unlikely that the observed association is entirely explained by residual confounding given the large magnitude of effect. Second, it is probable that a single pooled effect estimate inadequately represents the true underlying effect given the considerable heterogeneity of the literature. Among the studies included in this review, there was a lack of reporting of patient characteristics and a large degree of heterogeneity remains unexplained. It is therefore likely that the true underlying effect differs across some of the unreported demographic, behavioral, and treatment variables. Third, the results from this review may not be generalizable to individuals receiving more modern treatment modalities or to older adults diagnosed with lymphoma as most individuals in the evidence base were treated for lymphoma in the 20th century and were diagnosed with lymphoma before the age of 40.

## 5 CONCLUSIONS AND FUTURE DIRECTIONS

We have identified areas of future research based on the limitations of the evidence base. Future studies should attempt to control for potential confounders in addition to age, sex, and ethnicity. Investigators are encouraged to use directed acyclic graphs and g‐methods to separate the potential time‐varying confounding effects of psychosocial and lifestyle variables. In addition, future studies should strive to report a greater number of pre‐ and post‐treatment patient characteristics (eg, obesity, tobacco use, the presence of comorbidities), disease features (eg, subtype of Hodgkin and non‐Hodgkin lymphoma), and treatment variables (eg, the median treatment era, the proportion who received anthracyclines) with a higher level of granularity. Authors are also encouraged to assess and report on the extent to which loss‐to‐follow‐up may have biased their estimates and to consider exploring modification by age at diagnosis, stage, and prior history of cardiovascular disease. Lastly, the majority of research to date has focused on individuals diagnosed with lymphoma during childhood or young adulthood and there is a need for additional research that focuses on persons diagnosed with lymphoma later in life.

While clarifying the independent relation between prior lymphoma diagnosis and cardiovascular disease is important, the evidence to date suggests that patients with a history of lymphoma are at heightened risk for cardiovascular mortality. Even if the magnitude of the associations documented within this meta‐analysis are influenced by residual confounding and other sources of bias, healthcare providers should recognize that persons with a prior history of lymphoma, particularly if diagnosed with Hodgkin lymphoma before the age of 21, are at an increased risk of adverse cardiovascular outcomes. These findings highlight the importance of implementing cardiovascular surveillance, prevention, and screening interventions in lymphoma survivors.

## CONFLICT OF INTERESTS

Doreen M. Rabi received travel reimbursement from Hypertension Canada. Matthew T. James has an investigator initiated grant funded by Amgen Canada. No other potential conflict of interests are declared.
