# Supplementary material for: Long‐term risk of cardiovascular mortality in lymphoma survivors: A systematic review and meta‐analysis
Source: Cancer Med. 2018 Aug 15;7(9):4801–13. doi: 10.1002/cam4.1572 (PMC6143935; doi:10.1002/cam4.1572)
Supplement: Supplementary file 3 [file CAM4-7-4801-s003.docx]

Supplemental Table 1. Systematic Search Strategy used to Identify Relevant Articles Indexed in MEDLINE, EMBASE, and CINAHL Databases

|  | **Lymphoma** | **Survivor** | **Observational Study^1^** | **Cardiovascular Disease** |
| --- | --- | --- | --- | --- |
| **Title /Abstract Keywords** | 1. (lymphoma* or  Hodgkin*) | 3. (surviv* or remission or ((previous* or past or prior or histor*) adj5 (treat* or diagnos*))) | 5. (cohort* or (case* and control*) or cross-sectional or longitudinal or follow-up or prospective or retrospective or incidence or prevalence or risk or long-term or (late adj5 (effect* or risk* or onset))) | 7. ((cardi* adj5 (injur* or event* or disease* or mortality or morbidity or outcome* or arrest or death* or infarction)) or (heart adj5 (disease* or failure or attack)) or (valv* adj5 (disease* or abnormal*)) or (arter* adj5 disease*) or stroke* or angina* or CVD or "myocardial infarction" or thrombosis or tachycardia or atherosclerosis or cardiomyopathy or arrhythmia or carditis or aneurysm* or (late adj5 (mortality or morbidity)) or (long-term adj5 (mortality or morbidity)) or (cause-specific adj5 mortality)) |
| **MeSH Terms** | 2. exp lymphoma/ [Medline and Embase]  2. (MH "Lymphoma+") [CINAHL] | 4. exp survivors/ or exp survival rate/ [Medline]  4. exp survivor/ or exp/ survival [Embase]  4. (MH "Survivors+") [CINAHL] | 6. exp Epidemiologic Studies/ or exp Matched-Pair Analysis/ or exp Control Groups/ or exp Prevalence/ or exp Incidence/  6. exp cohort analysis/ or exp longitudinal study/ or exp prospective study/ or exp/ retrospective study/ or exp case control study/ or exp cross-sectional study/ [Embase]  6. (MH "Case Control Studies+") OR (MH "Prospective Studies+") OR (MH "Retrospective Design+") OR (MH "Incidence") OR (MH "Prevalence") OR (MH "Cross Sectional Studies") [CINAHL] | 8. exp cardiovascular diseases/ [Medline]  8. exp cardiovascular disease/ [Embase]  8. (MH "Cardiovascular Diseases+") [CINAHL] |
| **Combined Terms** | 9. 1 or 2  10. 3 or 4  11. 5 or 6  12. 7 or 8  13. 9 and 10 and 11 and 12 | | | |

^1^ The search terms used to identify relevant study designs were based on two previously published filters for observational studies [ref. 32,33].

NOTE: Search was limited to studies indexed before November 22^nd^, 2016
