# Supplementary material for: Long‐term risk of cardiovascular mortality in lymphoma survivors: A systematic review and meta‐analysis
Source: Cancer Med. 2018 Aug 15;7(9):4801–13. doi: 10.1002/cam4.1572 (PMC6143935; doi:10.1002/cam4.1572)
Supplement: Supplementary file 4 [file CAM4-7-4801-s004.docx]

Supplemental Table 2. Newcastle Ottawa Scale (NOS) Study Quality Assessment

| **Ref.** | **First Author (Year)** | **Selection Score^1^** | **Comparability Score** | **Outcome Score** | **Total Score** |
| --- | --- | --- | --- | --- | --- |
| [46] | Henry-Amar (1990) | 2 | 1 | 1 | 4 |
| [47] | Hancock (1993a) | 3 | 1 | 2 | 6 |
| [48] | Hancock (1993b) | 3 | 2 | 1 | 6 |
| [49] | Robertson (1994) | 3 | 1 | 2 | 6 |
| [50] | Mauch (1995) | 3 | 1 | 1 | 5 |
| [51] | King (1996) | 3 | 2 | 2 | 7 |
| [53] | Glanzmann (1998) | 3 | 1 | 3 | 7 |
| [52] | Brierley (1998) | 3 | 1 | 3 | 7 |
| [54] | Hudson (1998) | 2 | 1 | 2 | 5 |
| [56] | Reinders (1999) | 3 | 1 | 3 | 7 |
| [57] | Shah (1999) | 3 | 1 | 2 | 6 |
| [55] | Green (1999) | 3 | 1 | 2 | 6 |
| [59] | Eriksson (2000) | 3 | 1 | 1 | 5 |
| [58] | Avilés (2000) | 2 | 1 | 3 | 6 |
| [60] | Lee (2000) | 3 | 1 | 3 | 7 |
| [61] | Avilés (2001) | 2 | 1 | 2 | 5 |
| [62] | Ng (2002) | 3 | 1 | 2 | 6 |
| [63] | Aleman (2003) | 3 | 1 | 3 | 7 |
| [64] | Avilés (2005) | 3 | 1 | 2 | 6 |
| [65] | Swerdlow (2007) | 2 | 1 | 3 | 6 |
| [66] | Mertens (2008) | 3 | 1 | 3 | 7 |
| [67] | Kiserud (2010) | 3 | 1 | 2 | 6 |
| [68] | Prasad (2012) | 3 | 1 | 3 | 7 |
| [69] | Kero (2015) | 3 | 1 | 2 | 6 |
| [70] | Bhuller (2016) | 3 | 1 | 3 | 7 |
| [71] | Henson (2016) | 3 | 1 | 3 | 7 |
| [72] | Fidler (2017) | 3 | 1 | 3 | 7 |

^1^ No studies excluded individuals with a history of cardiovascular disease at baseline
