# Supplementary material for: Long‐term risk of cardiovascular mortality in lymphoma survivors: A systematic review and meta‐analysis
Source: Cancer Med. 2018 Aug 15;7(9):4801–13. doi: 10.1002/cam4.1572 (PMC6143935; doi:10.1002/cam4.1572)
Supplement: Supplementary file 5 [file CAM4-7-4801-s005.docx]

Supplemental Table 3. Stratified Meta-Analyses of the Long-Term Risk of Cardiovascular Disease Mortality among Lymphoma Survivors

| **Strata** | **N^1^** | **SMR (95% CI)^2^** | **I^2** | **Q-Statistic** | **P-value^3^** |
| --- | --- | --- | --- | --- | --- |
| **Hodgkin** |  |  |  |  |  |
| **Age at diagnosis** |  |  |  |  |  |
| < 21 years | 11 | 13.43 (9.22 to 19.57) | 78.9% | Q = 47.28; p < 0.001 | 0.001 |
| ≥ 21 years | 4 | 3.33 (2.54 to 4.35) | 86.5% | Q = 22.22; p < 0.001 |  |
|  |  |  |  |  |  |
| **Sex** |  |  |  |  |  |
| Males | 12 | 4.73 (3.52 to 6.35) | 87.1% | Q = 85.47; p < 0.001 | 0.65 |
| Females | 10 | 4.19 (2.46 to 7.15) | 82.5% | Q = 51.48; p < 0.001 |  |
|  |  |  |  |  |  |
| **Follow-Up** |  |  |  |  |  |
| < 10 years | 6 | 3.52 (2.36 to 5.27) | 79.8% | Q = 24.77; p < 0.001 | 0.55 |
| 10 to < 15 years | 6 | 3.04 (2.11 to 4.37) | 65.3% | Q = 14.42; p = 0.013 |  |
| 15 to < 20 years | 5 | 4.06 (2.75 to 5.99) | 70.7% | Q = 13.65; p = 0.008 |  |
| ≥ 20 years | 5 | 4.49 (3.20 to 6.30) | 57.0% | Q = 9.30; p = 0.054 |  |
|  |  |  |  |  |  |
| **Treatment Regimen** |  |  |  |  |  |
| Radiation Only | 6 | 5.04 (2.89 to 8.81) | 88.3% | Q = 42.78; p < 0.001 | 0.73 |
| Radiation and Chemotherapy | 6 | 6.07 (3.34 to 11.04) | 83.3 | Q = 30.00; p < 0.001 |  |
|  |  |  |  |  |  |
| **Treatment Era** |  |  |  |  |  |
| < 1980 | 4 | 4.18 (3.12 to 5.45) | 83.7% | Q = 18.36; p < 0.001 | 0.36 |
| ≥ 1980 | 3 | 3.11 (1.88 to 5.14) | 87.8% | Q = 16.22; p < 0.001 |  |
|  |  |  |  |  |  |
| **Non-Hodgkin** |  |  |  |  |  |
| **Age at diagnosis** |  |  |  |  |  |
| < 21 years | 6 | 6.23 (3.35 to 11.59) | 63.0% | Q = 13.53; p = 0.019 | 0.97 |
| ≥ 21 years | 2 | 6.61 (0.45 to 96.59) | 98.6% | Q = 70.57; p < 0.001 |  |

^1^ Number of studies reporting stratum-specific estimate

^2^ Stratum-specific pooled standardized mortality ratio estimate (95% confidence intervals) from random-effects model

^3^ P-value corresponds to the significance of strata indicator variable(s) in a meta-regression model
