# Supplementary material for: Long‐term risk of cardiovascular mortality in lymphoma survivors: A systematic review and meta‐analysis
Source: Cancer Med. 2018 Aug 15;7(9):4801–13. doi: 10.1002/cam4.1572 (PMC6143935; doi:10.1002/cam4.1572)
Supplement: Supplementary file 6 [file CAM4-7-4801-s006.docx]

Supplemental Figure 1. Cumulative meta-analysis of the long-term risk of cardiovascular disease mortality among Hodgkin lymphoma survivors

Supplemental Figure 2. Funnel plot for assessment of publication bias among investigations of the long-term risk of cardiovascular mortality among lymphoma survivors
